# Behaviour in captivity predicts some aspects of natural behaviour, but not others, in a wild cricket population

**DOI:** 10.1098/rspb.2015.0708

**Published:** 2015-06-22

**Authors:** David N. Fisher, Adèle James, Rolando Rodríguez-Muñoz, Tom Tregenza

**Affiliations:** 1 Centre for Ecology and Conservation, University of Exeter, Penryn Campus, Penryn TR10 9FE, UK; 2 UFR Sciences et Techniques, Université François Rabelais, Parc Grandmont, Tours 37200, France

**Keywords:** animal personality, shyness, exploration, *Gryllus*, laboratory, wild

## Abstract

Examining the relevance of ‘animal personality’ involves linking consistent among- and within-individual behavioural variation to fitness in the wild. Studies aiming to do this typically assay personality in captivity and rely on the assumption that measures of traits in the laboratory reflect their expression in nature. We examined this rarely tested assumption by comparing laboratory and field measurements of the behaviour of wild field crickets (*Gryllus campestris*) by continuously monitoring individual behaviour in nature, and repeatedly capturing the same individuals and measuring their behaviour in captivity. We focused on three traits that are frequently examined in personality studies: shyness, activity and exploration. All of them showed repeatability in the laboratory. Laboratory activity and exploration predicted the expression of their equivalent behaviours in the wild, but shyness did not. Traits in the wild were predictably influenced by environmental factors such as temperature and sunlight, but only activity showed appreciable within-individual repeatability. This suggests that some behaviours typically studied as personality traits can be accurately assayed in captivity, but the expression of others may be highly context-specific. Our results highlight the importance of validating the relevance of laboratory behavioural assays to analogous traits measured in the wild.

## 1. Introduction

Individuals of the same species in the same population can show consistent among-individual differences in behaviour across time and contexts [1,2]. These apparent constraints on behavioural flexibility, termed ‘personality’, have been shown to be pervasive throughout animal taxa [3], with a range of evolutionary and ecological consequences for individuals and populations [4,5]. The study of consistent among-individual differences in behaviour provides an avenue for understanding apparently non-adaptive behaviour (e.g. pre-copulatory sexual cannibalism [6]) as well as some of the persistent variation around adaptive peaks of behaviour [7]. The study of animal personality has undergone a recent and rapid expansion, bringing difficulties associated with young disciplines, with many different definitions and techniques for answering related questions [8]. Nevertheless, it is widely recognized that studying animal personality in the wild is vital [9,10]. Studies of personality in nature allow for assessment of relevant effects on fitness [11], and have the potential to identify laboratory artefacts due to artificial responses to unnatural stimuli [10] and to differences in the expression of behaviour in wild and laboratory contexts [12].

It remains difficult to monitor the behavioural variation of individual animals in the wild, due to the workload required to capture, tag, release, and then reliably track and monitor individuals in their natural habitat (but see [13–18]). Rather than directly assay animals in the wild, many studies (but see [13,14,19,20]) capture wild animals, conduct standardized behavioural assays in captivity, and then release them and monitor life histories and fitness traits [21–30]. This is potentially problematic if personality assays in the laboratory do not reflect behaviour in the wild, or if single/short-term assays in a novel environment do not accurately reflect an individual's behavioural type for the trait of interest [31,32] (see [33,34] for further discussion). What we think is a measure of exploratory behaviour in the laboratory could actually be more analogous to susceptibility to anxiety in the wild; for example, an individual moving among many sections of an arena/box/tank as an anxious response to a novel environment [8]. This could lead to incorrect conclusions in relation to predictions of particular hypotheses (e.g. the existence of a relationship between fitness and exploratory behaviour in certain environments) [29]. Furthermore, the artificial stimulus presented in captivity might fall outside the range of stimuli normally experienced by the individual, giving it questionable relevance for understanding adaptive behaviour [10,12]. Despite this, few studies have related captive measures of personality to wild measures of the same personality traits in the same individuals. Herborn *et al.* [13] confirmed that in blue tits (*Cyanistes caeruleus*), captive measures of personality (neophobia and exploratory tendency) reflected analogous measures in the wild. North American red squirrels (*Tamiasciurus hudsonicus*), which scored highly for a composite measure representing activity and exploration, were subsequently trapped at more different locations in the field than those with low scores [35]. Contrastingly, Siberian chipmunks (*Tamias sibiricus*) that scored highly on an activity–exploration composite measure were trapped more often than less active/exploratory individuals, but not at a greater diversity of traps, suggesting they were less trap-shy and/or more active, but were not exploring the environment more [36]. Also with slightly conflicting results, van Overveld & Matthysen [37] found that fast-exploring great tits (*Parus major*) did not increase their home range more than slow-exploring individuals did when food availability changed, but did increase the distance they travelled to visit feeders. However, the latter result could be confounded by dispersal status, as immigrants both travelled further and had higher exploration scores than locally born individuals [37]. Others have looked for concordance among different traits (e.g. willingness to approach a mirror in captive tests and sociability in the wild [38–40], or shyness in captivity and activity in the wild [41]).

A further deficiency is that the majority of studies comparing individual behaviour between laboratory and wild contexts have been conducted on mammals and birds. In the one study on invertebrates, Briffa *et al.* [42] measured the same individual European hermit crabs (*Pagurus bernhardus*) for startle response in the wild and across four laboratory observations. They found significant concordance across these five tests, but did not directly test for repeatability across wild and laboratory settings [42]. Invertebrate personality especially has attracted a large amount of interest [43,44] and, for behaviours other than courtship, has been shown to be more repeatable than vertebrate behaviour [3]. However, studies on among-individual variation in invertebrate behaviour are almost completely restricted to captivity (but see [19,45]). Their small size, and the difficulty in tagging soft-bodied animals or those that regularly moult, contribute to this. Bell *et al.*'s meta-analysis [3] found wild behaviour to be more repeatable than laboratory behaviour. Therefore, one might expect wild invertebrate behaviour to be highly repeatable, as found in wild beadlet anemones (*Actinia equina*) [19], although more studies are required to confirm this as a general rule. Ultimately, we need to relate studies in the laboratory to studies in the wild in order to understand the behaviour of animals in their natural environment [46]. What we are lacking are direct and repeated measures of the same trait in both the wild and the laboratory in a range of taxonomic groups.

To this end, we repeatedly measured shyness (here defined as aversion to risk [47]), activity (general movement about an environment) and exploration (willingness to explore new areas of an environment [2]) in the wild in a population of field crickets (*Gryllus campestris*). During the same period, we repeatedly captured the same individuals and measured these three traits in captivity before releasing them. We also investigated what biotic (e.g. age and sex) and abiotic (e.g. weather and temperature) factors influenced wild behaviour. Finally, we quantified the importance of an individual's microhabitat at the point of measurement for its shyness. This allows us to determine whether an individual's habitat choice could influence its observed personality [19,48].

## 2. Material and methods

### (a) Data collection

Data were collected from April to July 2013 at a field site in northwest Spain, where we study a wild population of *G. campestris* as part of the long-running WildCrickets project (see [46] and www.wildcrickets.org for further details). In this species, nymphs and adults spend most of their lives in or around burrows that they individually dig in the ground and defend from other conspecifics. At our study site, eggs hatch from May to July and nymphs remain active until October–November, when they start diapause as late instars. They become active again between late January and March, when they start to forage and undergo one or two more nymphal moultings before they moult into adults in April–May. Once they become sexually mature, the males will sing to attract females, and both sexes will start moving frequently among burrows in search of mates. Males and females will share a burrow, but within sexes there are fights for possession of burrows and no co-habiting [49]. We caught adult crickets between 2 and 4 days after they emerged (3.76 ± 2.81, mean ± s.d.), and fixed a vinyl tag with cyanoacrylate glue with a unique alpha-numeric code. This method is the outcome of many years of testing; only 1–4% of crickets per year need to be re-tagged due to losing their original tag. We used a trap designed by Luke Meadows (https://crickettrapping.wordpress.com/). This is very effective at catching any cricket that attempts to emerge from the burrow, reducing the effect of trap-shy or trap-happy individuals [50,51]. Each cricket burrow is also individually identified with a unique three digit number. We placed 120 motion sensitive cameras at random among those burrows with an active cricket. These allow us to record behaviour in the natural setting and responses to stimuli. Crickets regularly move among burrows, giving a degree of independence between burrow ID and cricket ID.

### (b) Wild shyness

Between 8 May 2013 and 29 June 2013, on days when the weather was suitable for the crickets to be active (more than 14°C and not raining; around 90% of days during the 2013 field season), we carried out a disturbance trial by walking among the cameras in the field. When walking towards a cricket, the experimenter simulates an approaching predator and triggers the cricket's flight response into its burrow. As we passed each camera, we waved a paint brush briefly in front of it. This allows us to re-watch the video, and associate a cricket entering its burrow with the disturbance caused by ourselves rather than from another source. We scored shyness in the wild by measuring the time in seconds between the end of the disturbance (the brush last appearing on-screen) and the cricket re-emerging from its burrow. If there was a male–female pair at a burrow (5% of instances), we recorded this fact, but a shyness score was only assigned to the cricket emerging from the burrow first.

### (c) Wild activity and exploration

For our measures of activity, we recorded the total number of times a cricket left the area under observation by the camera over a burrow each day or part of a day it was under observation. Crickets are counted as leaving a burrow if they move out of view of the camera and do not return within 5 min. Therefore, the measure of activity is the number of these leaving events (‘leaves’) per day. The measure of exploration is the number of unique burrows a cricket is observed at per day (or part of a day) when it was under observation. Therefore, all behaviours in the wild were measured relatively continuously across the field season.

### (d) Laboratory behaviours

We recorded our initial measures of behaviour in the laboratory on crickets we caught for tagging, but prior to the tagging procedure. Once released after tagging, we re-caught crickets every 10–20 days from the first test until the cricket was no longer observed, to repeat the behavioural assays. This is a long enough gap to prevent habituation [52]. Therefore, the laboratory measures were collected regularly alongside the continuously collected wild measures. Our laboratory measure of shyness was the time to emerge from a refuge, an assay that has been used extensively in captive crickets [53–56], including by ourselves in this species (see [52] for further details). In a temperature-controlled room (19 ± 0.64°C; mean ± s.d.), we recorded the number of seconds for a cricket to emerge from a plastic tube, after the lid had been removed. This was achieved by the use of fixed cameras connected to a computer running iCatcher software (iCode Systems; http://www.icode.co.uk/icatcher/). Activity was scored as the rate of movement around a 290 × 201 × 212 mm plastic box, once the cricket had emerged from the tube. For exploration, we counted the number of unique virtual tripwires (set to measure activity, possible scores range from 0 to 8) the cricket crossed in the first minute after it emerged from the tube. See [52] for further details of the laboratory assays.

Before tests, crickets were held in standardized 150 ml plastic containers for at least 15 min while they were weighed. Tests ran for 30 min, after which the crickets were returned to their plastic collection containers. They were held in isolation in the same 150 ml plastic container for 30 min, and then the assay was repeated. For those not emerging from the tube within 30 min, the observation for all behaviours was recorded as missing (i.e. ‘n.a.’). If the cricket emerged from the tube in both tests on a particular day, only the first score was used. Less than 5% of crickets that left the refuge did so in the final 5 min of the trial, so extending the trial would not have allowed us to include many of the excluded individuals. Furthermore, giving these individuals an arbitrarily low/high score (e.g. zero activity or 30 min for shyness) would have created a large spike in the frequency of low/high values, making the distributions unamenable to analyses as otherwise they followed Poisson distributions. Finally, if an individual did not emerge from the tube in two trials and we gave it exactly the same score in each trial, this would artificially boost the calculated repeatability of behaviour as the cricket would appear perfectly consistent, and so increase the potential for type 1 statistical errors. Following the two tests, we returned crickets to the burrows they were captured from, which we kept blocked while the cricket was out of the field (typically 90–180 min) to prevent the burrow being occupied by another individual.

### (e) Data analysis

We conducted all analyses in R v. 3.1.2 [57]. For shyness in the wild, we removed any disturbances where the cricket was already inside the burrow long before the brush appeared on screen. Crickets did not emerge from the tube in the laboratory in 41% of trials, in which case the trial was recorded as a missing observation.

### (f) Relating wild and laboratory behaviours

We constructed separate generalized linear mixed models for each behaviour in the R package MCMCglmm [58]. These models have each laboratory behaviour score as a fixed effect, and that individual's next measured score for that behaviour in the wild as the response variable, following Herborn *et al.* [13]. A significant, positive effect of the laboratory behaviour on the wild behaviour indicates the two assays correspond to the same trait. We only used one wild behaviour measure for each laboratory score, so if two wild measures were recorded after a laboratory measure, only the first one was used. Furthermore, we never used a laboratory measure on the same day as a wild measure. We also included the number of days between the laboratory and the wild scores as a fixed effect, as well as the interaction between this interval and the laboratory behaviour score. We were interested in whether the interaction was significant (as our analyses were in a Bayesian framework, here ‘significant’ is used as a synonym for important) as this would show whether the ability of the laboratory measure to predict the wild measure depended on the timespan between them. For activity and exploration, we also included the number of minutes that crickets were under observation for the scored day as a fixed effect. We used a Poisson error structure, with additive errors and a log-link function for all models. We also included random effects of individual identity and, for wild shyness, burrow number. This also allows us to control for multiple measures per individual and per burrow. Adjusted repeatability (*R*_Ad_) for behaviours in the laboratory was calculated using all the data for the laboratory assays in three separate GLMMs, with laboratory behaviour as the response, age, temperature and sex as fixed effects, and individual identity as a random effect. The *R*_Ad_ measure is (having corrected for additional variables) the proportion of total variance that is reproducible among repeated measures of a certain group, and is also referred to as the intra-class correlation coefficient, in this instance the group/class being a single individual [59]. We calculated *R*_Ad_ of the wild behaviours using all data collected with multiple environmental covariates as the fixed effects (described in the electronic supplementary material). For the number of samples, number of unique individuals and the mean, standard deviation and range of number of tests per individual for each analysis, see electronic supplementary material, table S1.

We also implemented a bivariate approach, where behaviours in each context are two responses, and we tested for significant covariance. This gave equivalent results to the above method, but due to additional complexities, we do not discuss it any further (see the electronic supplementary material for more details). The methods for the statistical analysis of the behaviours in the wild are also available in the electronic supplementary material.

### (g) Interpreting results

Each of the effects is modelled as a distribution of the likely influence of that effect, defined by a mode (PDM) and its lower and upper 95% credible intervals (LCRI and UCRI, respectively). Fixed effects are judged significant if the estimates of the CRIs do not cross zero, while MCMCglmm also provides the pMCMC score, useful to assess ‘significance’ in analogy with studies using a frequentist framework. Random effects are measures of variance and so (except in unusual circumstances [60]) are always above zero. Therefore, their importance is judged by comparison with the residual variance and by calculating *R*_Ad_ scores with CRIs estimates of their own. *R*_Ad_ scores can be compared between models to see if traits are more or less repeatable than others. Model selection cannot be based on information criteria as the deviance information criterion calculated by MCMCglmm is not ‘focused’ appropriately for non-Gaussian data [61], nor can models be compared with *F* or likelihood ratio tests as the effects do not have effect sizes. Instead, we constructed the model with all terms of interest and interpreted the effect of each variable in the maximal model (following convention, e.g. [62]).

## 3. Results

### (a) Repeatability of laboratory behaviours

In the model with laboratory shyness as the response, the PDM of the among-individual variance was 0.30 (LCRI = 0.18, UCRI = 0.47), the PDM of the residual variance was 0.90 (LCRI = 0.76, UCRI = 1.0) and the *R*_Ad_ estimated to be 0.25 (LCRI = 0.16, UCRI = 0.36). For laboratory activity, the PDM of among-individual variance was 2.09 (LCRI = 1.29, UCRI = 3.20), the PDM of the residual variance was 4.63 (LCRI = 3.80, UCRI = 5.48) and the *R*_Ad_ was estimated as 0.32 (LCRI = 0.21, UCRI = 0.42). Laboratory exploration was similarly repeatable to the other traits; the PDM of the among-individual variance was 0.69 (LCRI = 0.41, UCRI = 1.13), the PDM of the residual variance was 1.01 (LCRI = 0.72, UCRI = 1.32) and *R*_Ad_ was estimated to be 0.29 (LCRI = 0.19, UCRI = 0.41). These scores are similar to the repeatability scores of traits in Bell *et al.*'s meta-analysis (mean: 0.37) [3], indicating that they represent individual-specific behaviours.

### (b) Relationships between laboratory and wild behaviours

**Figure 1.**
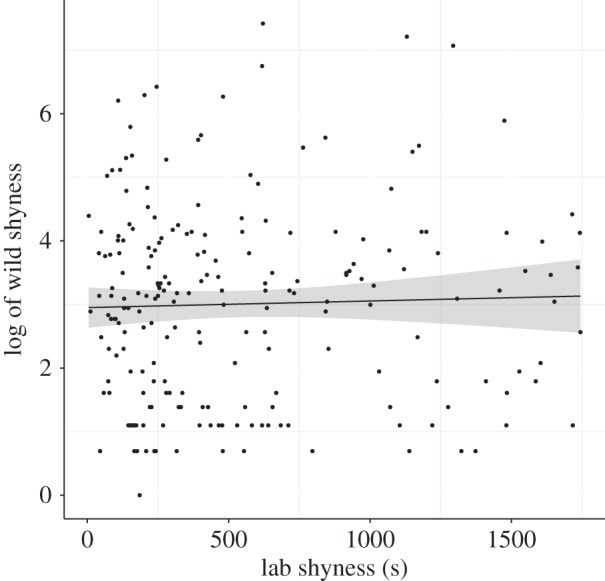
Laboratory shyness and log of wild shyness (logged to aid viewing). The line is from a simple linear model of wild shyness and laboratory shyness, the grey area indicates the standard errors around the estimate. There was no relationship between shyness in the laboratory and shyness in the wild (PDM ± 95% CRIs = −4.37 × 10^−5^ ± −6.08 × 10^−4^−7.36 × 10^−4^).

Laboratory shyness scores did not influence the wild shyness scores (figure 1; PDM = −4.37 × 10^−5^, LCRI = −6.08 × 10^−4^, UCRI = 7.36 × 10^−4^, pMCMC = 0.87). The interaction between laboratory shyness and the timespan between the laboratory and wild shyness scores was not important (PDM = 1.31 × 10^−5^, LCRI = −1.40 × 10^−4^, UCRI = 1.49 × 10^−4^, pMCMC = 0.90). The timespan between the laboratory and wild shyness scores did not influence the wild shyness score (PDM =−2.82 × 10^−2^, LCRI = −9.10 × 10^−2^, UCRI = 1.29 × 10^−1^, pMCMC = 0.82).

**Figure 2.**
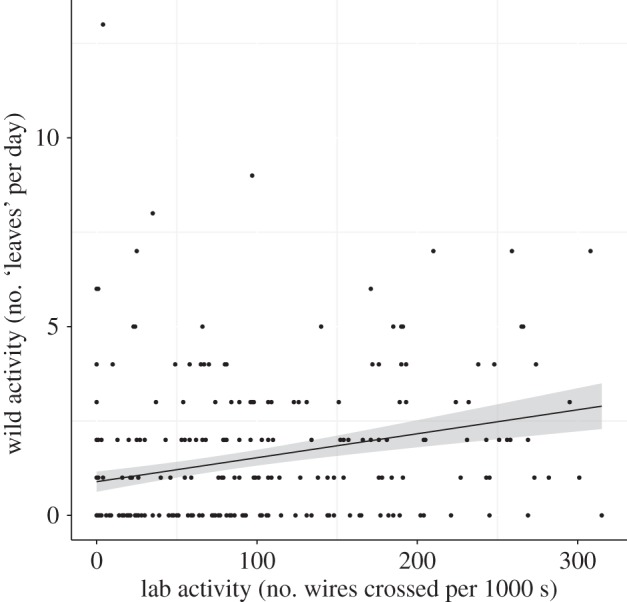
Laboratory activity and wild activity. The line is from a simple linear model of wild activity and laboratory activity, the grey area indicates the standard errors around the estimate. There was a significant, positive relationship between activity in the laboratory and activity in the wild (PDM ± 95% CRIs = 5.98 ± 3.99−8.89).

Laboratory activity level was positively related to level of activity in the wild (figure 2; PDM = 5.98, LCRI = 3.99, UCRI = 8.89, pMCMC < 0.01). The CRIs for interaction between the laboratory score and the time between the measures marginally overlapped zero (interaction: PDM = −0.99, LCRI = −2.18, UCRI = 0.09, pMCMC = 0.08), while the gap between measures negatively influenced wild activity level (PDM = −0.08, LCRI = −0.17, UCRI = −1.79 × 10^−3^, pMCMC = 0.04). The number of minutes a cricket was observed outside its burrow negatively influenced its activity score (PDM = −3.00 × 10^−4^, LCRI = −7.06 × 10^−4^, UCRI = −1.65 × 10^−5^, pMCMC = 0.04).

**Figure 3.**
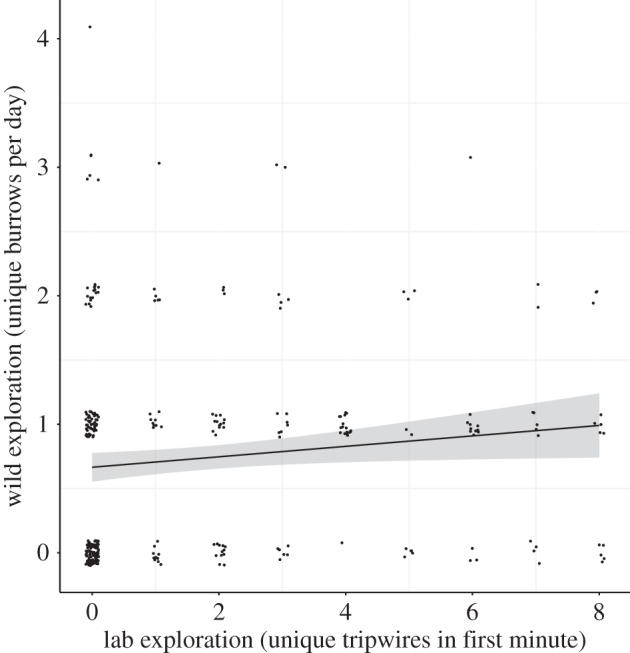
Laboratory exploration and wild exploration. The line is from a simple linear model of wild exploration and laboratory exploration, the grey area indicates the standard errors around the estimate. There tended to be a positive relationship between exploration in the laboratory and exploration in the wild (PDM ± 95% CRIs = 0.08 ± −0.01−0.15). Points are offset on both *x*- and *y*-axes to aid viewing when there are multiple points with the same *x* and *y* scores, which were always integers.

Exploration in the laboratory tended to positively predict exploration in the wild, although this was not significant at the 95% level (figure 3; PDM = 0.08, LCRI = −0.01, UCRI = 0.15, pMCMC = 0.07). The interaction between laboratory score and time between measures, and time between measures alone, were not important (interaction: PDM = −0.02, LCRI = −0.07, UCRI = 0.01, pMCMC = 0.26; time: PDM = −0.05, LCRI = −0.13, UCRI = 0.01, pMCMC = 0.11). The number of minutes a cricket was on-screen negatively influenced its exploration score (PDM = −4.50 × 10^−4^, LCRI = −7.90 × 10^−4^, UCRI = −2.10 × 10^−4^, pMCMC < 0.01).

Factors predicting behaviours in the wild, as well as the effect of microhabitat on the measurement of shyness, are presented in the electronic supplementary material, tables S2–S4, with the sample sizes in the electronic supplementary material, table S1.

To summarize, shyness in the laboratory and the wild were not related, but activity was positively associated between the two contexts, and exploration tended to be. All traits showed an appreciable degree of repeatability in the laboratory (*R*_Ad_ = 0.25, 0.32 and 0.29 for shyness, activity and exploration, respectively). Shyness was not at all repeatable in the wild (*R*_Ad_ = 0.06). Activity, and to a lesser degree exploration, showed a modest degree of repeatability in the wild (*R*_Ad_ = 0.21 and 0.12, respectively). Based on whether the 95% CRIs of the estimates of *R*_Ad_ overlapped or not, repeatability of shyness and exploration were significantly higher in the laboratory than the wild, and not different between the laboratory and the wild for activity. All behaviours in the wild could be predicted by various biotic and abiotic factors (see the electronic supplementary material).

## 4. Discussion

### (a) Relating captive and wild behaviours

We found a relationship between individuals' activity and exploration in the laboratory and the wild, but no such relationship for shyness. For shyness, this suggests that either we have in fact measured two different and unrelated traits, or that expression of this behaviour is highly context-specific [63]. Shyness measured in the laboratory showed a similar degree of repeatability as recorded in the literature for many other personality traits [3]. The significantly lower repeatability of shyness in the wild suggests that shyness expression in the wild is context-specific. Therefore, the natural setting has a high ‘situational strength’ for crickets (i.e. it has a strong influence on behaviour and so masks among-individual differences) [64]. This is not a general rule for poikilotherms; however, beadlet anemones (*A. equina*) show a high repeatability of startle response in the wild [19]. Shyness in the laboratory may therefore reflect responses to the stress of the artificial situation, rather than behavioural tendencies on a bold–shy continuum. The stimulus for the shyness test was necessarily different between the laboratory and the wild, as we could not bring each cricket's burrow into the laboratory. Such compromises will be necessary for many species when moving from the wild to the laboratory, although in some cases stimuli can be replicated (e.g. [42]). The fact that such a low *R*_Ad_ was observed in the wild and a typical *R*_Ad_ for a behaviour observed in captivity indicates that studying among-individual behavioural differences may be more viable in tightly controlled conditions for some traits.

Unlike shyness, activity showed a relationship between laboratory and wild measures, and equal repeatability in both contexts. Activity could be viewed as a more fundamental property of an animal's behaviour, reflective of differences in basal metabolic rate (BMR) [65] rather than more complex combinations of cost–benefit trade-offs. BMR commonly shows large intra-specific variability [65], and for activity it is easy to see how consistent differences in individual BMR could lead to consistently different levels of activity across contexts. It is interesting that our measures of activity in the two contexts were quite different (movement around a box in less than 30 min, and movement to and from burrows over the course of an entire day), yet still showed a strong relationship. Clearly, assays designed to test the same fundamental trait in different environments can achieve the same goal. There was also a tendency for a larger gap between assays to decrease the strength of the relationship. This suggests that within-individual change in behaviour over time can reduce the ability to detect relationships between contexts.

Exploration showed a weaker relationship between laboratory and wild measures, with a significantly lower repeatability in the wild than in the laboratory. Exploratory behaviour may be a more complex trait than activity, reflecting trade-offs a cricket makes based on its current condition, goals/requirements and the environmental conditions. This may have weakened our ability to capture individual differences in the wild, while we were able to detect greater individual differences in the laboratory. However, we still found a positive relationship, despite our assays of exploration being quite different. In particular, laboratory exploration was measured over only 1 min, yet was still related to exploration in the wild, which was measured over an entire day.

Ultimately, these results indicate the need to be careful when relating personality traits measured in the laboratory to traits in the wild, as a relationship might exist for some traits but not others. Researchers need to either validate their measurements by comparison with an analogous behaviour in the wild, or make every effort to ensure they are not in fact measuring something else (e.g. a stress response).

### (b) Predictors of wild shyness

The factors predicting shyness in the wild were time of day, sex and a weather–age interaction. Time of day was weakly and positively related to the delay before a cricket re-emerged from its burrow. Field crickets move less among burrows at night, probably as a result of the decreased ambient temperature [66], and possibly because of the threat of shrew and hedgehog predation. Therefore, as dusk approaches the advantage of ceasing daily activity and retreating into a safe refuge increases. Male crickets re-emerged from their burrows more quickly than female crickets. Males need to be outside of the burrow to sing in order to attract mates, which, depending on male size, increases reproductive success [46]. The correlation between number of mates and number of offspring in wild *G. campestris* is higher in males than females [46]. Therefore, males may benefit more from leaving a burrow to sing and potentially attract new mates than a female does in leaving the burrow to find new mating partners. Finally, there was a significant age–weather interaction, with older crickets re-emerging in sunny weather less quickly than expected. When young, adult crickets need to bask often to accelerate their development to sexual maturity (typically, 3–6 days post-emergence [67]). However, once sexual maturity is reached, the energy from the sun might accelerate the aging process, making individuals less willing to be exposed. In cloudy weather, this factor is removed, so young individuals have less need to emerge from the burrow.

The low estimate of *R*_Ad_ for wild shyness score indicates that shyness of wild crickets is dictated by external conditions (some of which we identify here) rather than by the identity of the individual. This contrasts with the beadlet anemone (*A. equina*), which showed a high level of *R*_Ad_ for a startle response [19]. However, Briffa & Greenaway [19] pointed out that unmeasured aspects of the microhabitat (e.g. position in the pool or exposure to currents and predators) could be important for sedentary species such as anemones. Indeed, in the laboratory, where these factors are absent, *A. equina* show intermediate levels of *R*_Ad_ [68]. Briffa & Greenaway [19] also note that repeatability of analogous behaviours can vary greatly among invertebrate phyla, suggesting that we need further work to understand why, in the field, crickets (*G. campestris*) show low repeatability in willingness to take risks (this study), hermit crabs (*P. bernhardus*) show intermediate consistency [42] and anemones (*A. equina)* show high consistency [19]. This could be due to the mobility of the animals in question. Mobile species that experience a range of conditions might show low repeatability in the wild, while more sedentary species with a stable microhabitat might show high repeatability. It has also been suggested that among-individual behaviour variation is non-adaptive, and instead arises from constraints in development [69]. Whether taxon-specific developmental pathways lead to these differences should be investigated.

### (c) Microhabitat and shyness

Accounting for microhabitat only slightly reduced our estimate of individual *R*_Ad_ for shyness in the wild (from 0.10 to 0.06; see the electronic supplementary material), with the 95% CRIs of these scores overlapping substantially. There was also little variance in shyness attributed to among-burrow differences. This suggests that either all burrows are very similar or that the differences among them do not affect cricket behaviour. The crickets dig the burrows themselves in the autumn and spring [46], which allows them to choose a location and orientation. They also move among burrows throughout the course of the season, abandoning some and regularly using or digging others [46,66]. This allows them to move if the burrow they are using does not match their preferences, suggesting that differences among those burrows used by crickets do not have a great effect on this measure of shyness.

### (d) Predictors of wild activity and exploration

Crickets were more active and explored more when the sun was stronger, and less when it was raining. Crickets were unsurprisingly more active when it was warmer, but not more exploratory. Therefore, although temperature drives more general movement about the environment, it does not cause crickets to visit new areas. This also demonstrates the importance of direct sunlight, rather than simply ambient temperature, in influencing cricket behaviour. Females were more active than males. In this species, males typically sing at burrows to attract mates while females move among them, which may drive this difference in activity. The sexes were equally exploratory, however, so the higher activity shown by females was to repeatedly visit the same burrows, rather than to employ their additional activity to visit multiple different burrows and males in the same day. Females of this species benefit from mating multiply [46], but might visit males across different days rather than within the same day. Crickets were more active and exploratory when older. An increase in these traits with age was also found in the laboratory assays [52]. A lower residual reproductive value at old age may increase risk-taking behaviour, and so increase the willingness of a cricket to move around its environment to find mates [70,71]. Furthermore, older crickets were more active and exploratory when it was raining than younger crickets, perhaps as older crickets are more willing to take the risks involved in these conditions. Alternatively, older crickets might have a more robust physiology or be more highly chitinized, allowing them to move about the environment despite the rain. Males increased activity levels more when older, so the difference in activity between males and females was lower in older crickets. This might reflect the diminishing return for females in continually acquiring new mates, whereas for males the return is near-linear [72]. Males also increased their exploration more as they aged than females did, although this interaction was very weak. Crickets that were on-screen for longer, unsurprisingly, recorded higher activity levels, but did not record higher exploration levels. This probably results from the fact that a cricket can potentially move between neighbouring burrows in a few seconds, so visiting many burrows does not require being on-screen for a long period of time. Older crickets were less active than expected when it was sunny, and also less exploratory. This complements the finding that older crickets are slower to emerge from the burrow when it is sunny, perhaps as a response to the accelerating effects of sunshine on senescence processes. Finally, crickets were more active and exploratory later in the field season. This could be a response to the limited window in which to acquire mates, as at the end of the season in July there are very few other crickets alive. Individuals who are alive at later dates might need to be more active and exploratory to find mates.

### (e) Personality in the laboratory and wild traits

Previous work has found relationships between cricket personality in captivity and sexual signalling [55], immune response [53] and predation pressure [54]. Furthermore, work on laboratory personality measures in our study population has revealed relationships with aging [52]. This, along with relationships between laboratory and wild assays in two out of three of the traits we measured here, suggests that personality measured in the laboratory is not irrelevant to adaptation in the wild. For shyness at least, the laboratory can be said to have ‘low situational strength’, allowing among-individual differences to be detected [64]. However, it seems odd that an environment as unnatural as the laboratory would have such a low impact on cricket behaviour. Indeed, the trait we measured in the laboratory might not be the trait we thought it was at all. Crucially, to determine whether shyness (and activity and exploration) observed in the laboratory is relevant or unrelated to selective forces in nature, it must be compared with fitness-relevant traits of the same individuals in the wild (e.g. social interactions and life history). Previous work on various vertebrates has demonstrated relationships between personality traits measured in captivity and fitness [24,29], competitive ability [22], territoriality [25], social network position [21] and rate of promiscuity [30], although no relationship was found with environmental sensitivity [23] and only a weak relationship with BMR [27].

Alongside the ability to detect among-individual differences in a controlled environment, an additional strength of laboratory studies is the ability to conduct experimental manipulations to test hypotheses. Such manipulations are typically very difficult in the wild. However, in some systems, direct experimental manipulations in the wild are feasible. For example, in the field crickets, we could alter burrow characteristics such as grass cover to determine if crickets respond to the characteristics of their microhabitat with behavioural changes. The ability to perform a variety of experimental manipulations with limited resources is another advantage of studying invertebrates.

## 5. Conclusion

There were relationships between some behaviours we measured in the laboratory and their analogues in the wild. This cautions against assuming that ecologically relevant measures of personality can easily be made by removing animals from their natural context. Assays that appear superficially similar may in fact measure different dimensions of personality. Existing literature outlines a variety of relationships between captive personality assays and natural and sexual selection in wild vertebrates, while some types of study systems and questions will necessarily require that animals be brought into captivity. Nevertheless, every effort should be made to ensure that such assays are good proxies for the particular trait of interest as expressed in the wild.

## Supplementary Material

Supplementary materials

## Data accessibility

Data are available through Open Research Exeter (http://hdl.handle.net/10871/16930).
