# Supplementary material for: Behaviour in captivity predicts some aspects of natural behaviour, but not others, in a wild cricket population
Source: Proc Biol Sci. 2015 Jun 22;282(1809):20150708. doi: 10.1098/rspb.2015.0708 (PMC4590455; doi:10.1098/rspb.2015.0708)
Supplement: Supplementary materials [file rspb20150708supp1.docx]

# Supplementary Materials

Table S1. Table showing total number of samples, number of unique individuals and mean, standard deviations and ranges for frequency of tests per individual for each analysis of each behaviour carried out. The analyses modelling the trait in either the laboratory or the wild used every available measure for those contexts, while the lab vs. wild comparison used a laboratory measure and the subsequent wild measure. For shyness in the wild, the number of unique burrows is shown in brackets next to the number of unique individuals.

| Trait | Analysis | Samples | Unique individuals | Mean | S.D | Range |
| --- | --- | --- | --- | --- | --- | --- |
| Shyness | Lab vs. wild | 220 | 153 | 1.44 | 0.65 | 1-3 |
|  | Laboratory | 617 | 202 | 1.30 | 1.86 | 1-8 |
|  | Wild | 946 | 147 (156) | 5.28 | 4.25 | 1-16 |
| Activity & exploration | Lab vs. wild | 303 | 186 | 1.63 | 0.79 | 1-4 |
|  | Laboratory | 617 | 202 | 1.30 | 1.86 | 1-8 |
|  | Wild | 4087 | 229 | 17.9 | 11.9 | 1-50 |

Predictors of wild behaviour

To determine which quantifiable factors influence behaviour in the wild, irrespective of the measure in the laboratory, we constructed three separate GzLMMs, one for each trait. Every wild score for each particular trait was used as the response variable, and sex, age, date, weather and temperature were used as fixed effects. Weather and temperature were recorded by a weather station in the centre of the meadow that takes measurements every ten minutes (Vantage Pro 2, Davis instruments, California). For shyness, weather was either “sunny” or “cloudy” when the disturbance was carried out, while for activity and exploration weather was the separate (although not necessarily independent) effects of the mean amount of solar radiation and the total amount of rain on that day. Temperature (in the shade) was either the temperature at the record nearest the disturbance (for shyness, which was measured at a single point) or the mean temperature over the day (for activity and exploration, which were measured across the day). For shyness we also included the time and whether the cricket was with an individual of the opposite sex or not at the point of measurement as fixed effects. For activity and exploration we included the number of minutes the cricket was on-screen for that day as a fixed effect. All continuous fixed effects (minutes observed outside the burrow, age, date, time of day, temperature and weather for activity and exploration) were transformed by subtracting the global mean from each individual value and then dividing by the standard deviation [1,2]. This leaves each variable in the same units: “number of standard deviations the datum is above or below the mean”, common practice in selection analyses as it allows the effect/response of different factors to be directly compared [3,4].

We included interactions between sex and age, sex and weather (two separate interactions for the two weather components in the activity & exploration analyses) and, for shyness, sex and pair to determine whether the sexes differed in how they changed with age, how they responded to environmental conditions and if they responded differently to having a partner respectively. An interaction with sex and age might be expected if the sexes follow different reproductive strategies that require different rates of mate acquisition and so differences in willingness to leave the burrow and move about their environment [5]. A sex-weather interaction is plausible, as in this species males need to leave the burrow to sing to attract mates, a behaviour which is highly metabolically costly [6,7] and so is likely to be weather dependent. The sexes might differ in how they respond to being in a pair as male crickets have been shown to be “chivalrous”: when in a pair it is usually the female who stays nearer the burrow and enters it first when the pair are threatened by a predator [8]. We also included interactions between age and weather (again, two separate interactions for activity & exploration) and age and pair (only for shyness), to determine whether crickets of different ages respond differently to environmental conditions or being in a pair respectively. An age-weather interaction is likely as direct sunlight affects the rate of cricket development [9], so the time spent basking in sunshine is likely to change over a cricket’s lifetime. An age-pair interaction was included as residual reproductive value is lower in old age [10,11] and so could alter behaviour while with a mate in comparison to when alone. We included random effects of individual identity and, for shyness, burrow identity. Again for all models we used Poisson error structures with additive errors and log-link functions, and calculated R_Ad_ separately for individuals and burrows following Nakagawa & Schielzeth (2010). We included more fixed effects in this model than in the model comparing wild and laboratory behaviour as it has a substantially larger data set, the previous model only contained pairs of laboratory and wild measures on the same individual where the former preceded the latter.

To determine whether ignoring burrow identity (microhabitat) leads to an increased estimate of individual consistency, we removed the random effect of burrow from GLMM for shyness, and compared the new estimates of R_Ad_ with the original GLMM. The difference was considered important if the 95% CRIs of the estimate of R_Ad_ did not overlap.

Predictors of wild shyness

We recorded 946 measures for shyness in the wild, across 179 individuals and at 167 unique burrows. The results from the GzLMMs for the shyness in the wild are presented in table S2. Time of day was weakly and positively related to the time a cricket took to come out of its burrow. Male crickets were shyer than female crickets. Finally, there was a significant age-weather interaction, with older crickets coming out in sunny weather more slowly than younger crickets. R_Ad_ of individuals and burrows was both estimated to be 0.06 (LCRI = 0.04, UCRI = 0.12).

Table S2. Predictors of wild shyness. An effect is modelled as a distribution (the posterior distribution). Below we give the mode of that distribution (PDM) and the lower & upper 95% credible intervals (LCRI & UCRI, respectively). We also include the pMCMC scores, interpreted in a similar way to traditional p values. Sex is modelled with female as the default and males as the contrast and weather is modelled with “cloudy” as the default and “sunny” as the contrast.

| Factor | PDM | LCRI | UCRI | pMCMC |
| --- | --- | --- | --- | --- |
| Intercept | 3.068 | 2.801 | 3.332 | <0.001 |
| Sex | -0.379 | -0.805 | -0.072 | 0.028 |
| Age | -0.132 | -0.385 | 0.228 | 0.587 |
| Pair | 0.689 | -0.348 | 1.583 | 0.221 |
| Temperature | -0.063 | -0.221 | 0.065 | 0.315 |
| Weather | -0.072 | -0.367 | 0.274 | 0.804 |
| Date | -0.087 | -0.374 | 0.172 | 0.525 |
| Time | 0.116 | 0.016 | 0.252 | 0.032 |
| Sex:age | 0.039 | -0.121 | 0.361 | 0.409 |
| Sex:weather | 0.280 | -0.152 | 0.765 | 0.168 |
| Sex:pair | 0.041 | -0.895 | 1.486 | 0.726 |
| Age:weather | 0.350 | 0.104 | 0.571 | 0.005 |
| Age:pair | -0.061 | -0.605 | 0.342 | 0.654 |
| Among-individual variance | 0.203 | 0.112 | 0.375 | NA |
| Among-burrow variance | 0.197 | 0.111 | 0.352 | NA |
| Residual variance | 2.482 | 2.192 | 2.692 | NA |
| Repeatability of individuals | 0.061 | 0.040 | 0.120 | NA |
| Repeatability of burrows | 0.062 | 0.037 | 0.119 | NA |

Microhabitat and shyness

In the model for wild shyness the PDMs of among individual variance and among burrow variance were both 0.20 (table S2). The R_Ad_ of both individuals and burrows was 0.06. When the random effect of burrow was removed from the model, the PDM of the among-individual variance rose to 0.26 (LCRI = 0.17, UCRI = 0.46) and the R_Ad_ to 0.10 (LCRI = 0.06, UCRI = 0.16).

Predictors of wild activity

We recorded 4097 measures of activity in the wild for 229 individuals. The results are presented in table S3. Crickets were more active on sunnier and warmer days, as well as when they were older, whereas a lower activity was found on rainy days. Females were more active than males. There was a significant, positive age:rain interaction, so older crickets were more active and exploratory when it was raining than younger crickets. There was a significant, positive sex:age interaction; males increased activity levels more when older, so the difference in activity between males and females was lower in older crickets. Crickets that were on screen for longer recorded higher activity levels. The significant, negative age:sun interaction indicates that older crickets were less active than expected when it was sunny. Finally, crickets were more active at later dates. R_Ad_ of individuals was calculated to be 0.21 (LCRI = 0.17, UCRI = 0.27).

Table S3. Predictors of activity in the wild. An effect is modelled as a distribution (the posterior distribution), below we give the mode of the posterior distribution (PDM) and lower & upper 95% credible intervals (LCRI & UCRI respectively). We also include the pMCMC scores, interpreted in a similar way to traditional p values. Sex is modelled with female as the default and males as the contrast.

| Factor | PDM | LCRI | UCRI | pMCMC |
| --- | --- | --- | --- | --- |
| Intercept | 0.050 | -0.045 | 0.191 | 0.223 |
| Minutes | 0.117 | 0.084 | 0.167 | < 0.001 |
| Sex | -0.227 | -0.407 | -0.065 | 0.011 |
| Age | 0.135 | 0.031 | 0.238 | 0.014 |
| Temperature | 0.060 | 0.005 | 0.109 | 0.045 |
| Sun | 0.219 | 0.178 | 0.280 | < 0.001 |
| Rain | -0.172 | -0.225 | -0.090 | < 0.001 |
| Date | 0.447 | 0.351 | 0.580 | < 0.001 |
| Sex:age | 0.131 | 0.045 | 0.216 | 0.003 |
| Sex:sun | 0.032 | -0.049 | 0.114 | 0.416 |
| Sex:rain | 0.022 | -0.114 | 0.102 | 0.957 |
| Age:sun | -0.110 | -0.141 | -0.065 | < 0.001 |
| Age:rain | 0.089 | 0.047 | 0.160 | 0.001 |
| Among-individual variance | 0.248 | 0.200 | 0.356 | NA |
| Residual variance | 0.324 | 0.278 | 0.374 | NA |
| Repeatability | 0.210 | 0.169 | 0.267 | NA |

Predictors of exploration in the wild

For exploration we recorded 4097 measures of 229 individuals. The results are presented in table S4. Crickets explored more when older, when the sun was stronger and less when it was raining. Furthermore, there was a significant age:rain interaction, so older crickets were more exploratory when it was raining than younger crickets. The weak positive sex:age interaction indicates that males increased their exploration more as they aged than females did. Older crickets were less exploratory than expected when it was sunny, as indicated by the negative age:sun interaction. Finally, crickets were more exploratory at later dates. R_Ad_ of individuals was calculated to be 0.12 (LCRI = 0.08, UCRI = 0.15).

Table S4. Predictors of exploration in the wild. An effect is modelled as a distribution (the posterior distribution), below we give the mode of the posterior distribution (PDM) and lower & upper 95% credible intervals (LCRI & UCRI respectively). We include also the pMCMC scores, interpreted in a similar way to traditional p values. Sex is modelled with female as the default and males as the contrast.

| Factor | PDM | LCRI | UCRI | pMCMC |
| --- | --- | --- | --- | --- |
| Intercept | -0.335 | -0.432 | -0.260 | < 0.001 |
| Minutes | -0.024 | -0.067 | 0.012 | 0.236 |
| Sex | -0.080 | -0.209 | 0.041 | 0.184 |
| Age | 0.175 | 0.077 | 0.250 | 0.001 |
| Temperature | 0.011 | -0.044 | 0.062 | 0.835 |
| Sun | 0.265 | 0.217 | 0.321 | < 0.001 |
| Rain | -0.093 | -0.165 | -0.017 | 0.007 |
| Date | 0.307 | 0.217 | 0.397 | < 0.001 |
| Sex:age | 0.083 | 0.002 | 0.166 | 0.050 |
| Sex:sun | 0.038 | -0.045 | 0.112 | 0.482 |
| Sex:rain | -0.042 | -0.159 | 0.051 | 0.300 |
| Age:sun | -0.087 | -0.124 | -0.045 | < 0.001 |
| Age:rain | 0.101 | 0.033 | 0.150 | < 0.001 |
| Among-individual variance | 0.120 | 0.082 | 0.157 | NA |
| Residual variance | 0.045 | 0.033 | 0.059 | NA |
| Repeatability | 0.117 | 0.083 | 0.146 | NA |

# Bivariate analyses

As an alternative method to determine whether traits in the wild and the laboratory were related, we constructed a bivariate model for each trait. Entered as responses were every measure of the trait in the laboratory and every measure of the trait in the wild (see Table S1). The only fixed effects entered were sex and age of the individuals at the point of testing, as the other factors do not appear in both contexts. We initially included temperature in each context but these models failed to converge. For each record there is either a score for the laboratory behaviour or the wild behaviour, the other trait being recorded as a missing observation. Therefore, the residual covariance cannot be estimated so is fixed at zero.

Correlations are then calculated by dividing the covariance between the two responses by the square root of the product of their variances. This can be calculated across the posterior distributions of the variances and covariances, giving a mode and 95% CRIs for each correlation.

The results are presented in Tables S5-7. In summary they agree with the results of the analysis in the main text: shyness measures between the two contexts are not related, but activity and exploration are.

Table S5. Results of the bivariate model between laboratory and wild shyness. An effect is modelled as a distribution (the posterior distribution), below we give the mode of the posterior distribution (PDM) and lower & upper 95% credible intervals (LCRI & UCRI respectively). We also include the pMCMC scores, interpreted in a similar way to traditional p values. The intercept of wild shyness is modelled as a contrast to the global intercept i.e. of lab shyness. Sex is modelled with female as the default and males as the contrast.

| Trait | PDM | LCRI | UCRI | pMCMC |
| --- | --- | --- | --- | --- |
| Intercept | 5.766 | 5.629 | 5.933 | NA |
| Intercept – wild shyness | -1.431 | -1.621 | -1.258 | NA |
| Sex on lab shyness | -0.056 | -0.284 | 0.150 | 0.533 |
| Sex on wild shyness | -0.124 | -0.271 | 0.021 | 0.094 |
| Age on lab shyness | -0.220 | -0.303 | -0.165 | <0.001 |
| Age on wild shyness | 0.062 | -0.028 | 0.142 | 0.171 |
| V_A_ of lab shyness | 0.237 | 0.139 | 0.395 | NA |
| V_A_ of wild shyness | 0.029 | 0.015 | 0.063 | NA |
| CoV_A_ lab -wild shyness | 0.011 | -0.038 | 0.0643 | NA |
| Correlation lab -wild shyness | 0.287 | -0.309 | 0.631 | NA |

Table S6. Results of the bivariate model between laboratory and wild activity. An effect is modelled as a distribution (the posterior distribution), below we give the mode of the posterior distribution (PDM) and lower & upper 95% credible intervals (LCRI & UCRI respectively). We also include the pMCMC scores, interpreted in a similar way to traditional p values. The intercept of wild activity is modelled as a contrast to the global intercept i.e. of lab activity. Sex is modelled with female as the default and males as the contrast.

| Trait | PDM | LCRI | UCRI | pMCMC |
| --- | --- | --- | --- | --- |
| Intercept | 3.435 | 3.047 | 3.789 | NA |
| Intercept – wild activity | -3.457 | -3.861 | -3.197 | NA |
| Sex on lab activity | -0.103 | -0.735 | 0.294 | 0.446 |
| Sex on wild activity | -0.329 | -0.577 | -0.136 | 0.002 |
| Age on lab activity | 0.968 | 0.868 | 1.092 | < 0.001 |
| Age on wild activity | 0.676 | 0.623 | 0.738 | < 0.001 |
| V_A_ of lab activity | 2.977 | 2.301 | 4.055 | NA |
| V_A_ of wild activity | 0.499 | 0.386 | 0.658 | NA |
| CoV_A_ lab -wild activity | 0.666 | 0.451 | 0.997 | NA |
| Correlation lab -wild activity | 0.546 | 0.406 | 0.706 | NA |

Table S6. Results of the bivariate model between laboratory and wild exploration. An effect is modelled as a distribution (the posterior distribution), below we give the mode of the posterior distribution (PDM) and lower & upper 95% credible intervals (LCRI & UCRI respectively). We also include the pMCMC scores, interpreted in a similar way to traditional p values. The intercept of wild exploration is modelled as a contrast to the global intercept i.e. of lab exploration. Sex is modelled with female as the default and males as the contrast.

| Trait | PDM | LCRI | UCRI | pMCMC |
| --- | --- | --- | --- | --- |
| Intercept | 0.172 | -0.130 | 0.398 | NA |
| Intercept – wild exploration | -0.791 | -1.001 | -0.492 | NA |
| Sex on lab exploration | -0.115 | -0.475 | 0.238 | 0.564 |
| Sex on wild exploration | -0.169 | -0.372 | -0.031 | 0.026 |
| Age on lab exploration | 0.695 | 0.532 | 0.798 | < 0.001 |
| Age on wild exploration | 0.580 | 0.512 | 0.632 | < 0.001 |
| V_A_ of lab exploration | 0.705 | 0.442 | 1.256 | NA |
| V_A_ of wild exploration | 0.224 | 0.148 | 0.312 | NA |
| CoV_A_ lab -wild exploration | 0.267 | 0.135 | 0.432 | NA |
| Correlation lab -wild exploration | 0.652 | 0.389 | 0.840 | NA |

# References

1. Jackson, J. E. 2003 *A User’s Guide to Principal Components*. Hoboken, New Jersey: Wiley-Blackwell. [cited 2014 Mar. 3].

2. Van den Berg, R. A., Hoefsloot, H. C. J., Westerhuis, J. A., Smilde, A. K. & van der Werf, M. J. 2006 Centering, scaling, and transformations: improving the biological information content of metabolomics data. *BMC Genomics* **7**, 142. (doi:10.1186/1471-2164-7-142)

3. South, S. H., House, C. M., Moore, A. J., Simpson, S. J. & Hunt, J. 2011 Male cockroaches prefer a high carbohydrate diet that makes them more attractive to females: implications for the study of condition dependence. *Evolution* **65**, 1594–606. (doi:10.1111/j.1558-5646.2011.01233.x)

4. Hunt, J., Brooks, R. & Jennions, M. D. 2005 Female mate choice as a condition-dependent life-history trait. *Am. Nat.* **166**, 79–92. (doi:10.1086/430672)

5. Schuett, W., Tregenza, T. & Dall, S. R. X. 2010 Sexual selection and animal personality. *Biol. Rev. Camb. Philos. Soc.* **85**, 217–46. (doi:10.1111/j.1469-185X.2009.00101.x)

6. Mowles, S. L. 2014 The physiological cost of courtship: field cricket song results in anaerobic metabolism. *Anim. Behav.* **89**, 39–43. (doi:10.1016/j.anbehav.2013.12.014)

7. Hoback, W. W. & Wagner, W. E. 1997 The energetic cost of calling in the variable field cricket, Gryllus lineaticeps. *Physiol. Entomol.* **22**, 286–290. (doi:10.1111/j.1365-3032.1997.tb01170.x)

8. Rodríguez-Muñoz, R., Bretman, A. & Tregenza, T. 2011 Guarding males protect females from predation in a wild insect. *Curr. Biol.* **21**, 1716–9. (doi:10.1016/j.cub.2011.08.053)

9. Remmert, H. 1985 Crickets in Sunshine. *Oecologia* **68**, 29–33. (doi:10.1007/BF00379469)

10. Williams, G. 1966 Natural selection, the costs of reproduction, and a refinement of Lack’s principle. *Am. Nat.* **100**, 687–690.

11. Hirshfield, M. F. & Tinkle, D. W. 1975 Natural selection and the evolution of reproductive effort. *Proc. Natl. Acad. Sci.* **72**, 2227–2231. (doi:10.1073/pnas.72.6.2227)

12. Nakagawa, S. & Schielzeth, H. 2010 Repeatability for Gaussian and non-Gaussian data: a practical guide for biologists. *Biol. Rev. Camb. Philos. Soc.* **85**, 935–56. (doi:10.1111/j.1469-185X.2010.00141.x)
